# Bone Spheroid Development Under Flow Conditions with Mesenchymal Stem Cells and Human Umbilical Vein Endothelial Cells in a 3D Porous Hydrogel Supplemented with Hydroxyapatite

**DOI:** 10.3390/gels10100666

**Published:** 2024-10-18

**Authors:** Soukaina El Hajj, Martial Bankoué Ntaté, Cyril Breton, Robin Siadous, Rachida Aid, Magali Dupuy, Didier Letourneur, Joëlle Amédée, Hervé Duval, Bertrand David

**Affiliations:** 1 Laboratoire de Mécanique Paris-Saclay, CNRS, CentraleSupélec, ENS Paris-Saclay, Université Paris-Saclay, 91190 Gif-sur-Yvette, France; soukaina.el-hajj@centralesupelec.fr; 2 Laboratoire de Génie des Procédés et Matériaux, CentraleSupélec, Université Paris-Saclay, 91190 Gif-sur-Yvette, France; martial.bankoue-ntate@centralesupelec.fr (M.B.N.); cyril.breton@centralesupelec.fr (C.B.); magali.dupuy@centralesupelec.fr (M.D.); herve.duval@centralesupelec.fr (H.D.); 3 Laboratoire de Bioingénierie Tissulaire (BioTis), INSERM U1026, Université de Bordeaux, 33076 Bordeaux, France; robin.siadous@inserm.fr (R.S.); joelle.amedee@inserm.fr (J.A.); 4 Laboratoire de Recherche Vasculaire Translationnelle (LVTS), INSERM U1148, Université Paris Cité, 75018 Paris, France; rachida.aid@gmail.com (R.A.); didier.letourneur@inserm.fr (D.L.); 5 Laboratoire de Recherche Vasculaire Translationnelle (LVTS), INSERM U1148, Université Sorbonne Paris Nord, 93430 Villetaneuse, France

**Keywords:** bone regeneration, spheroids, porous hydrogel-based scaffolds, perfusion bioreactor, oxygen transport, spatial reorganization, 3D cell culture, Lattice Boltzmann method, fluid dynamics

## Abstract

Understanding the niche interactions between blood and bone through the in vitro co-culture of osteo-competent cells and endothelial cells is a key factor in unraveling therapeutic potentials in bone regeneration. This can be additionally supported by employing numerical simulation techniques to assess local physical factors, such as oxygen concentration, and mechanical stimuli, such as shear stress, that can mediate cellular communication. In this study, we developed a Mesenchymal Stem Cell line (MSC) and a Human Umbilical Vein Endothelial Cell line (HUVEC), which were co-cultured under flow conditions in a three-dimensional, porous, natural pullulan/dextran scaffold that was supplemented with hydroxyapatite crystals that allowed for the spontaneous formation of spheroids. After 2 weeks, their viability was higher under the dynamic conditions (>94%) than the static conditions (<75%), with dead cells central in the spheroids. Mineralization and collagen IV production increased under the dynamic conditions, correlating with osteogenesis and vasculogenesis. The endothelial cells clustered at the spheroidal core by day 7. Proliferation doubled in the dynamic conditions, especially at the scaffold peripheries. Lattice Boltzmann simulations showed negligible wall shear stress in the hydrogel pores but highlighted highly oxygenated zones coinciding with cell proliferation. A strong oxygen gradient likely influenced endothelial migration and cell distribution. Hypoxia was minimal, explaining high viability and spheroid maturation in the dynamic conditions.

## 1. Introduction

The need for bone substitutes has become of high relevance for musculoskeletal system-related injuries. In Europe alone, the cost of fractures has been estimated to be around EUR 37 billion [1]. In fact, they constitute at least 66% of significant physical traumas [2]. Although bone loss happens naturally with age, especially among a steadily growing elderly population, many conditions, such as autoimmune diseases, osteoporosis, menopause, and hormonal disorders, accelerate the process to a critical and sometimes fatal degree [3]. The alarming issue arises when these disorders, as well as others, cause non-union bone fractures that the body cannot heal nor seal on its own due to the size of the injury. This type of large bone defect requires surgical reconstruction using bone alternatives that can restore functionality to help avoid any possible amputation [4]. Current treatment plans use autologous, allogeneic, and xenogeneic grafts, as well as vastly different biomaterials. The gold standard in bone tissue transplantation is the autologous bone graft, meaning a graft that has been harvested from the patient, due to its low immunogenicity and high osteoinductive and osteoconductive properties [5]. Unfortunately, harvesting the tissue from the patient not only imposes a limit on the supply of grafts, which varies depending on the size of the injury, but it also requires an additional surgical procedure [6].

The limited supply imposed by autografts and allografts has urged medical practitioners and researchers to find innovative alternatives, with bone being the second most transplanted tissue after blood [7]. The knowledge of the different structural levels of bone and their highly mineralized nature allowed material engineers to design suitable alternatives that mimic the mechanical properties of this composite tissue. Nevertheless, in repair strategies for large bone defects, clinical issues seem to linger over the years as grafts commonly undergo necrosis due to the lack of integration with the host’s vasculature.

Many materials of both natural and synthetic origins, immobilized or not with bioactive agents, have been tested for their osteogenic abilities in vitro and in vivo. While some models made it past clinical testing to the medical market, they were still better suited for small bone defects. The solution for overcoming this conundrum lies with prioritizing vascular integration in the design of bone constructs. Ideally, following the implantation of scaffolds in vivo, two main vascular events must take place: macro-vasculature at the injury site allowing steady healing, and micro-vasculature at the scaffold level facilitating cell viability and therefore bone formation [8]. While there were initial attempts aimed at adding certain growth factors or endothelial precursor cells to induce angiogenesis in vivo, the resulting micro-vessels were often too small and insufficient to facilitate adequate integration. Micro-vessels were more prone for early regression and were usually restricted in length to only several micrometers per day. This would require weeks for the vessels to fully integrate within the implanted scaffolds; meanwhile, the accepted distance for the diffusion of oxygen and nutrients is within the range of 100–200 µm [9,10]. This prompted the emergence of pre-vascularization as a promising strategy, enabling the cultivation of a micro-vasculature in vitro across the scaffold prior to in vivo implantation. In the context of bone tissue engineering, it seems unmindful to omit endothelial elements when designing substitutes. One might argue that the involvement of endothelial cells in bone therapies is indispensable, irrespective of the need for pre-vascularization in in vivo constructs, owing to the synergistic relationship between blood and bone. The blood vessels structurally organize the architecture of bone tissue around them, while also simultaneously upholding bone homeostasis and facilitating the transportation of progenitor cells and growth factors [11].

The fundamental unit of blood vessels is the endothelial cell, which proliferates and migrates under the instructions and guidance of growth factors to give rise to complex vascularity. However, this physiological construction is codependent on the contribution and support of surrounding cells and structures, such as perivascular cells, to guarantee the establishment of functional vessels that are capable of sustaining proper blood circulation [12]. Within the interests of experimental co-culture designs, Mesenchymal Stem Cells (MSCs) serve a dual function by acting as osteoblast precursors and as perivascular-like cells that are capable of inducing and supporting angiogenesis [13,14]. The co-culture of endothelial cells and osteoblast precursors, be it direct or indirect, has been explored in research, proving its effectiveness in upregulating cell proliferation, bone formation, and/or vascularization, both in vitro and in vivo [15,16,17,18,19,20,21,22,23,24].

In the earlier and current stages of bone research, investigations into two-dimensional (2D) co-culture have shown enhanced cellular interactions and increased expression of angiogenic and osteogenic markers compared to monoculture groups [25]. Nevertheless, three-dimensional (3D) culture offers a far more advantageous and physiologically-relevant microenvironment, favoring cell-to-cell interaction and cell-to-matrix interaction, as well as the reorganization of cells, leading to vascular formation [26,27]. As 3D cell cultures better mimic the complex in vivo microenvironment found in tissues and organs, the spatial arrangement of cells in 3D also reflects, or implies, the natural organization of tissues. This spatial context is crucial for understanding cell behavior and interactions in response to external culture conditions. For regenerative medicine applications, a more controlled spatial distribution of cells within spheroids, and of spheroids within scaffolds, might positively influence tissue architecture, functionality, and integration with the host environment. Moreover, the spatial architecture of cells can impact essential processes such as proliferation and differentiation, which are highly relevant in the MSC culture [28,29]. While most of the literature available on spatial biology seems to specifically tackle in vivo embryological events, it still provides insight into the cell’s encoded genetic disposition. Although the impact of mechanics on mammalian cells is extensively documented, there is a dearth of research addressing the influence of oxygen on the spatial reorganization of cells during the maturation of spheroids or organoids. This gap is imposed by the challenges associated with obtaining accurate experimental measurements in 3D culture settings. At the scale of the spheroid, typically a few hundred micrometers, the real-time quantification of oxygen using external probes is experimentally challenging without perturbing the biological system and introducing possible contaminants. Thus, we propose here digital modeling and numerical simulations as alternative solutions for oxygen mapping, which can significantly enhance our understanding, albeit in an idealized manner, of the microenvironment within scaffolds [30].

In this study, we investigated the effects of initial cell seeding density (CSD) and perfusion flow rate on the co-culture of the MSC and the HUVEC spheroids in porous pullulan/dextran hydrogels. These scaffolds have been supplemented with hydroxyapatite particles to mimic the natural bone composition and provide necessary mechanical support and bioactivity that promote effective the osteo-differentiation of the MSCs [31,32,33]. Our main objective was to identify and analyze optimal 3D co-culture conditions by observing cell functionalities and employing computational fluid dynamics to visualize the oxygen transport and the shear stress forces generated by the perfusion system.

## 2. Results and Discussion

### 2.1. Structural Characterizations of 3D Scaffolds

#### 2.1.1. X-ray Microtomography of Hydrogels in a Dry State

**Figure 1:**
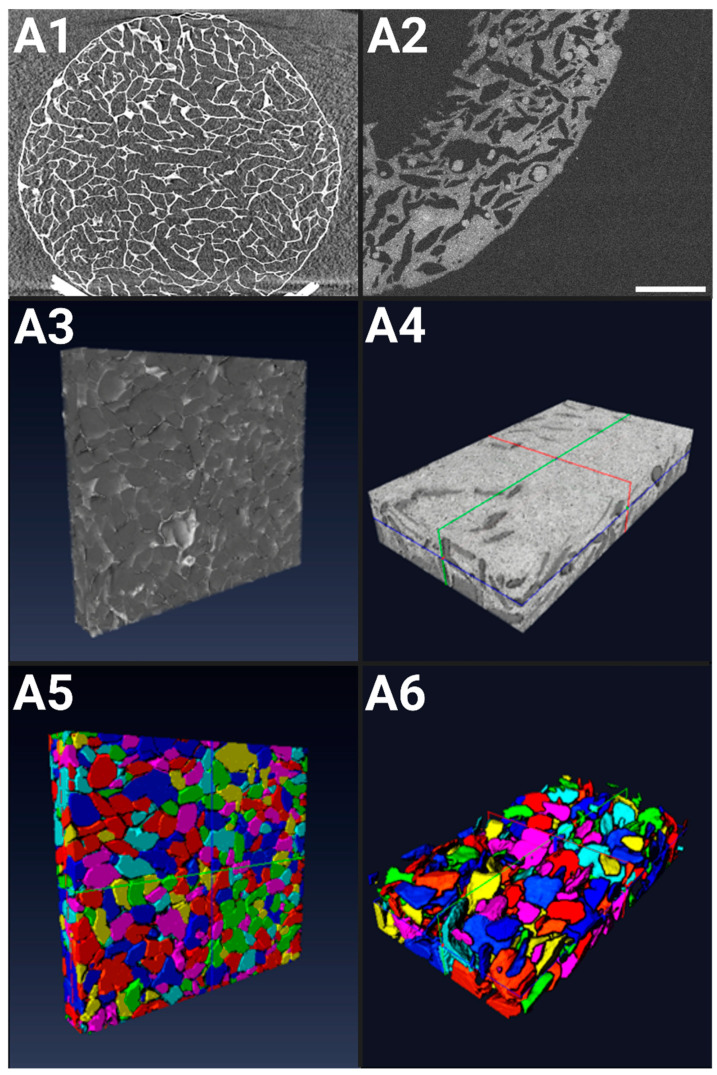
Characterization of hydrogels in the dry state: (**A1**) X-ray microtomography XY slice of a full hydrogel (8.6 mm in diameter, 1.8 mm in thickness), at 500 μm depth, voxel size of (8.4 μm × 8.4 μm × 8.4 μm); (**A3**) X-ray computed tomography 3D rendering of a scaffold ROI; (**A5**) Optical Coherence Tomography (OCT) 3D rendering of a scaffold ROI. In the hydrated state: (**A2**) OCT 2D rendering of a scaffold ROI showing seeded spheroids (dark white circles), scale bar = 1000 µm; (**A4**) OCT 3D rendering of a scaffold ROI; (**A6**) 3D rendering following pore labeling and reconstruction of the scaffold center. (**A3**,**A5**) ROI (5.6 mm × 0.9 mm × 6.4 mm), voxel size of (0.92 μm × 0.92 μm × 0.92 μm); (**A4**,**A6**) ROI (4.9 mm × 4.9 mm × 0.3 mm), voxel size of (8 μm × 8 μm × 1.45 μm).

The architecture of freeze-dried hydrogels in their dry state was examined using X-ray microtomography, featuring a voxel size of 8.4 μm × 8.4 μm × 8.4 μm. Following 3D image reconstruction, the resulting acquisitions revealed a highly porous structure with thin walls (Figure 1(A1)). The dry pores appeared polygonal in shape, likely due to the freeze-drying process involving polygonal-shaped ice crystals and appeared to be uniformly distributed along the z-axis. Notably, the surface porosity closely resembled that observed at the center of the dry hydrogel.

After reconstructing the 3D acquisitions (Figure 1(A3,A5)), the images were treated and labeled for a proper analysis, revealing a porosity of ε = 68% ± 1% in the dry state. The pore sizes ranged from 5 to 850 μm, with a mean pore size (cubic root of pore volume) of 254 ± 8 μm. Micropores (<100 μm) constituted approximately 12% of the total, while macropores (>100 μm) comprised the remaining 88%, according to the standard classification (ASTM F2450 18, 2018).

#### 2.1.2. OCT of Hydrogels in a Hydrated State

After the initial cell seeding, the hydrogels remain in a hydrated state until the end of the experiment. OCT was used to capture the porous structure of the hydrated scaffolds (Figure 1(A2)), achieving an axial Z resolution of 1.45 μm and an XY spatial resolution of (8 μm × 8 μm). The porosity of the hydrogels in their hydrated state varies along the Z-direction depth. Near the surface (up to 150 μm below the surface), porosity is low, contrasting with the higher porosity observed at the center of the hydrogel. Overall, the image analysis revealed a mean porosity of ε = 25% ± 4%.

The images were processed to allow the optimal segmentation of the hydrogel’s porous network, revealing a mean pore size (cubic root of pore volume) of 108 ± 10 μm (Figure 1(A4,A6)). Pore sizes within the hydrated scaffold ranged from 0 to 300 μm. The swelling of the scaffold due to hydration in the culture medium resulted in a reduction in pore size, notably pronounced at the surface of the hydrogel. Consequently, the mean pore size decreased 2.8-fold compared to the hydrogels in the dry state.

In the context of bone tissue engineering, early investigations indicated that a minimum pore size of 75–100 µm is necessary for substantial bone growth, with an optimal range of 100–135 µm. Subsequent studies have emphasized the importance of pores larger than approximately 300 µm for vascularization and bone ingrowth, while pores smaller than 300 µm may promote osteochondral ossification [34,35]. More recently, the in vivo culture of BM-MSCs in scaffolds of varying average pore sizes (173 µm, 275 µm, 384 µm) showed a significant increase in the expression of COL1, OPN, and OCN in the group with the smallest pore size [36]. The freeze-drying protocol produces hydrogels with an average pore size of 170 µm in a hydrated state, which fits well within the recommended dimensions to optimize osteogenesis [37,38]. The addition of hydroxyapatite particles (6.3% *w*/*w*) to the formula enhanced the osteoconductive profile of the hydrogel but led to a decrease in the average pore size to 100 µm, which still falls within the optimal range. Highly porous and permeable materials are particularly effective in facilitating the release and circulation of proteins, genes, or chemical signals, strengthening cellular communication and stimulation in the process [39]. While higher porosity leads to increased proliferation rates in vitro, it suppresses osteogenic factors such as ALP and osteocalcin; the opposite behavior is observed in vivo [40]. As our hydrogels have a porosity rate of 68% in dry state and 25% in hydrated state, this presents a good compromise between porosity and mechanical integrity which is essential for bone substitutes. Moreover, our co-culture system extends beyond osteogenic modeling to integrate angiogenic precursors that also command their own peculiar mechanical needs. This level of porosity and interconnectivity should allow the progression and the infiltration of a vasculogenic network, leading to scaffold pre-vascularization [41].

### 2.2. Characterizations of MSC/HUVEC Spheroids Within Hydrogels

#### 2.2.1. Spheroid Mapping

**Figure 2:**
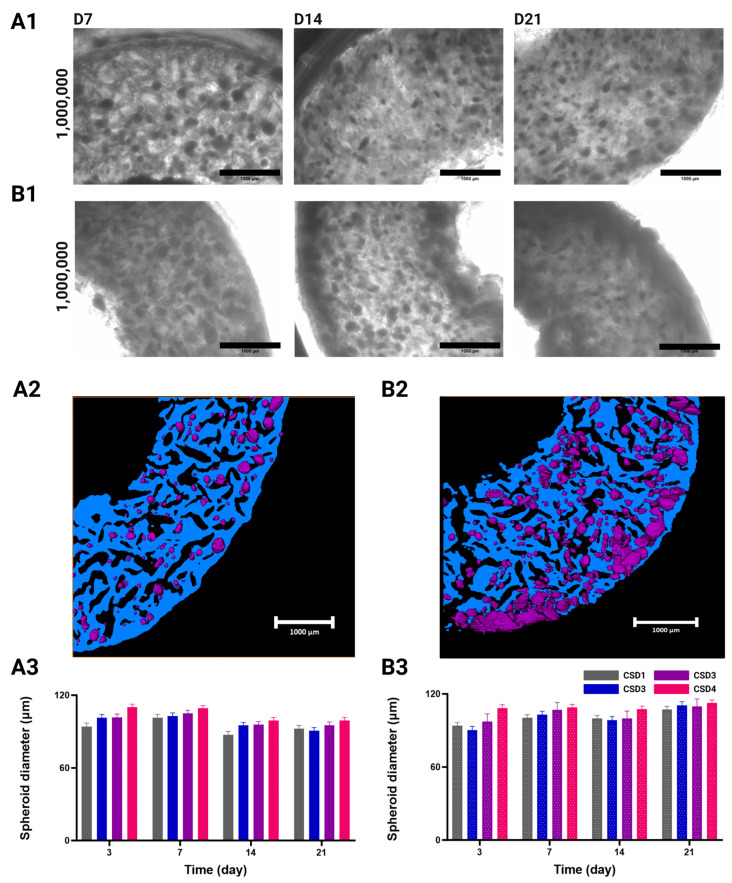
White field acquisitions of seeded hydrogel sections, showing the distribution of MSC/HUVEC spheroids in (**A1**) static culture conditions and (**B1**) dynamic culture conditions in a perfusion bioreactor, with CSD4 (1,000,000 cells/hydrogel scaffold). Scale bar = 1000 µm. Acquisitions of remaining cell seeding densities are found in the Supplementary Materials. Segmented OCT acquisitions showing spheroid (in purple) topology and distribution in scaffolds (in blue) with CSD4 (1,000,000 cells/hydrogel scaffold) (**A2**) At 24 h post implantation, and after (**B2**) 21 days of dynamic culture conditions. Scale bar = 1000 µm. Evolution of the MSC/HUVEC spheroid diameter (mean + SEM µm) throughout the culture period under (**A3**) static culture conditions and (**B3**) dynamic culture conditions. Cell cultures were conducted with CSD1 (400,000 cells/hydrogel scaffold), CSD2 (600,000 cells/hydrogel scaffold), CSD3 (800,000 cells/hydrogel scaffold), and CSD4 (1,000,000 cells/hydrogel scaffold); dynamic culture results are represented with a pattern (░).

The shape and distribution of formed aggregates throughout 21 days of culture, under either dynamic or the static conditions, was first assessed using brightfield microscopy (Figure 2(A1,B1) and Figure S1) and OCT (Figure 2(A2,B2)). Acquisitions were segmented along the XY directions to delineate the distribution of spheroids within hydrogels.

Under static culture conditions, spheroids were found to be randomly distributed within the porous network of the hydrogel, with their total number increasing proportionally to the cell seeding density (CSD) (Figure 2(A1) and Figure S1A). A similar effect was seen under dynamic culture conditions during the first two weeks of culture (Figure 2(B1) and Figure S2B). These hydrogels do not possess any adhesion sites (albeit they can be altered to have them), which normally impedes uniform spheroid formation [42]. Therefore, the resulting homogeneity in spheroid distribution following the cell seeding is an advantageous accolade to have in a macroporous hydrogel. Our seeding protocol allows the cells to spatially arrange themselves in situ according to their physiological tendencies and in response to their surrounding biochemical environment. This macroporous scaffold guides the cell aggregation through the shape and interconnectivity of its pores, but does not dictate their intercellular communication as it lacks a thoroughly defined ECM network [43].

After 3 weeks, we observed the formation of a dense and dark layer at the outer periphery of the hydrogel scaffold in comparison to the other regions in the CSD1 and CSD2 samples (Figure S1B). This area forms a crown of approximately 460 ± 50 µm in thickness. For CSD3 and CSD4, the phenomenon was amplified at the external periphery and was observed at earlier times on the internal periphery of the hydrogel (Figure 2(B1) and Figure S1B). Respectively, the outer and inner zones display crowns of approximately 500 ± 50 µm and 465 ± 100 µm in thickness.

With the OCT image reconstruction, it was revealed that these masses are cellular in nature, conforming to the contours of the hosting pores as the spheroids have proliferated enough to occupy the available space around them (Figure 2(B2)).

#### 2.2.2. Spheroid Diameter

To deepen our understanding of the molding effect that the hydrogel’s morphology has on the aggregation process of cells, we assessed the evolution of the spheroid average diameter as a function of the initial CSD and culture conditions (Figure 2(A3,B3)).

The growth patterns of the selected spheroids were shown to be dependent on the CSD, culture condition, and time, but not in a statistically significant manner. However, delving into such findings can be important for understanding how the culture environment influences the spheroid’s development. With respect to CSD, there was a slight increase in spheroid size with an average spheroid diameter across time and the culture conditions of 96 ± 1 µm for CSD1, 98 ± 1 µm for CSD2, 98 ± 1 µm for CSD3, and 102 ± 1 µm for CSD4.

Furthermore, a growth in spheroid size was detected throughout time under the dynamic culture conditions. Across all the CSDs, we measured a 1.1-fold increase in the average spheroid diameter from 97 ± 3 µm on D3 to 110 ± 3 µm on day 21. It should also be noted that the peripheral growth masses were omitted from this assessment as they possessed highly irregular shapes due to an increase in cell proliferation and/or the fusion of neighboring spheroids. No such growth was detected in the static conditions.

Despite the effects of culture conditions on the evolution of diameter throughout the culture period, the spheroids remained adherent to the average pore size of the hydrogels (108 ± 10 μm in hydrated state). However, it is important to note that spheroid size does not necessarily reflect the spheroid’s density and volume. Further, the spheroids cultured in the static conditions primarily exhibited irregular edges after 2 weeks of culture, indicative of a loosely packed architecture. Nevertheless, the porous scaffolds indeed served as templates or molds for cell aggregates by providing a conducive 3D environment that supported cell proliferation and organization into tissue-like structures. This also further proves the structural integrity of our scaffold as it preserved its architectural dimensions even after 21 days of dynamic culture in a perfusion bioreactor.

#### 2.2.3. Spheroid Volume

Hydrogels possess retention and saturation limits of cells per volume. Such limits can result in varying aggregation patterns and sizes of spheroids depending on the initial CSD without necessarily adhering to a linearly proportional model. Using OCT, we analyzed the resulting spheroids with increasing CSDs 24 h post-seeding (Table 1). The spheroids were segmented, labeled, reconstructed in 3D, then analyzed in Avizo version 2019.1. First, a spatial point pattern analysis revealed a strongly uniform distribution of spheroids along the 3 axes, mirroring the pattern observed in pore distribution. This suggests a degree of controllability over the seeding process, a desirable feature that promotes repeatability. Second, as the CSD increased, so did the size and quantity of the formed spheroids. Notably, this trend was disrupted slightly in the case of CSD3, where smaller spheroid volumes were observed compared to CSD2. However, this was counterbalanced by an exceedingly high number of spheroids. Overall, there was a 1.8-fold increase in both average spheroid volume and total spheroid number between CSD4 and CSD1, indicating a nearly linear growth pattern with respect to the initial CSD.

### 2.3. Proliferation and Viability of the MSC/HUVEC Spheroids

#### 2.3.1. Cell Proliferation Quantification

**Figure 3:**
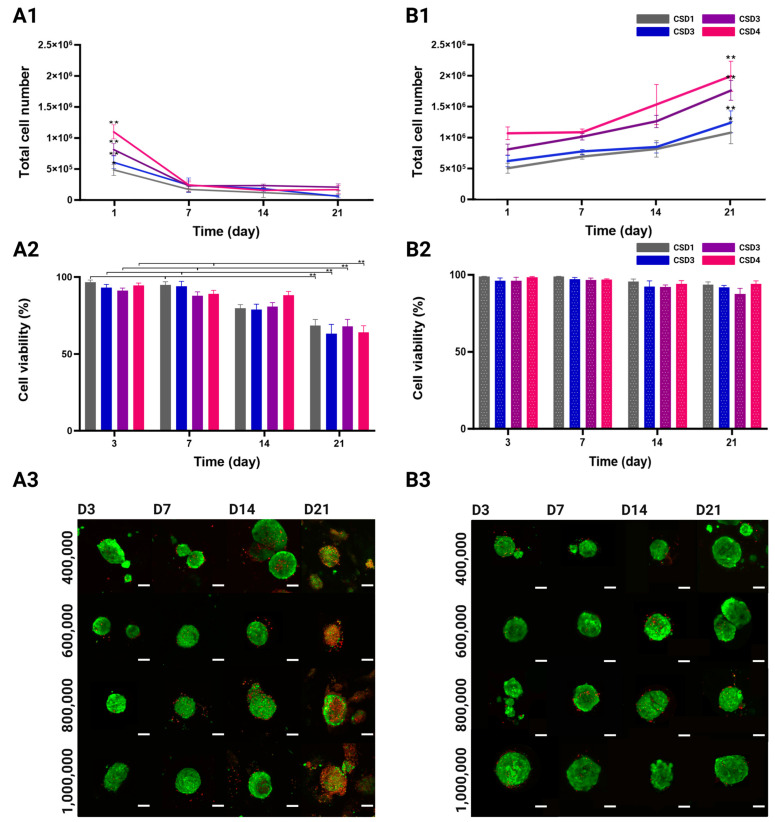
Total number of cells in MSC/HUVEC co-culture over 21 days. (**A1**) Hydrogels in static culture conditions, and (**B1**) in dynamic culture conditions. Evolution of MSC/HUVEC viability (mean + SEM %) using a LIVE/DEAD kit. Cells were cultured in (**A2**) static conditions *N* = 224, and (**B2**) dynamic conditions. Cell cultures were conducted with CSD1 (400,000 cells/hydrogel scaffold), CSD2 (600,000 cells/hydrogel scaffold), CSD3 (800,000 cells/hydrogel scaffold), and CSD4 (1,000,000 cells/hydrogel scaffold); dynamic culture results are represented with a pattern (░). * and ** denote *p* < 0.05 and *p* < 0.01, respectively. 2D representative projections of confocal z-stack acquisitions showing the distribution of viable cells (calcein-AM, green) and dead cells (ethidium homodimer-1, red) in the co-culture spheroids under (**A3**) the static culture conditions, and (**B3**) the dynamic culture conditions. Scale bar = 100 µm.

After 1 week of culture under the static conditions, the total cell number per hydrogel quickly and significantly fell by 2.1-fold for CSD1 and CSD2, 2.7-fold for CSD3, and 3.4-fold for CSD4 (Figure 3(A1)). In contrast, proliferation rates were different in the groups that were subjected to perfusion flow, especially in the second and third weeks of culture, where a growth spurt took place across all the CSDs (Figure 3(B1)). We detected a significant increase of 1.4-fold and 2.0-fold in the total cell number per hydrogel.

It should be noted that the compactness of the spheroids rendered it extremely difficult to separate the cell types in order to measure their individual growth patterns. Hence, these quantitative CyQUANT™ kit results reflect the total cell number of both the HUVECs and the MSCs, inseparably.

#### 2.3.2. Viability Quantification and Mapping

High cellular viability directly reflects optimal 3D co-culture conditions as they must provide cells with adequate oxygen and nutrient delivery among many other factors. Following the same experimental design, viability was observed and quantified in all the culture conditions and cell densities over 21 days of culture. The confocal images show a qualitative difference in cell viability between the static and the dynamic culture groups (Figure 3(A3,B3)). For the first 2 weeks of culture, the spheroids in all densities and culture conditions were predominantly viable (green) with occasional dead cells (red) more commonly found at the center of the spheroid. On day 21, a visible decrease in viability was detected under the static culture conditions as the red ethidium homodimer-1 staining became more prevalent. On the other hand, the spheroids maintained their viable appearance in the dynamic conditions, even after 3 weeks of culture.

The quantitative analysis of the confocal acquisitions showed no variation across different cell seeding densities in terms of viability (Figure 3(A2,B2)). Viability rates in the static culture conditions significantly decreased from 97 ± 1% for CSD1, 93 ± 2% for CSD2, 91 ± 2% for CSD3, and 95 ± 2% for CSD4 on day 3 to 68 ± 4%, 63 ± 6%, 68 ± 5%, and 64 ± 4%, respectively, by the end of the culture period. On average, there was a 1.4-fold decrease in viability by day 21. It is also important to highlight that this decline in viability was not sudden but gradual throughout time.

The dynamic culture was much more favorable in maintaining cell viability. A minimal decline in viability rates was observed from 99 ± 1% for CSD1, 96 ± 3% for CSD2, 96 ± 2% for CSD3, and 96 ± 1% for CSD4 on day 3 to 94 ± 5%, 92 ± 6, 93 ± 6%, and 94 ± 4%, respectively, by day 21.

It should also be noted that LIVE/DEAD staining does not make it possible to distinguish the viability of each cell type, but it can be safely assumed that our assessment is relevant for both the HUVECs and the MSCs.

### 2.4. 3D Spatial Reorganization of the MSCs and the HUVECs

#### 2.4.1. Reorganization of Cells

**Figure 4:**
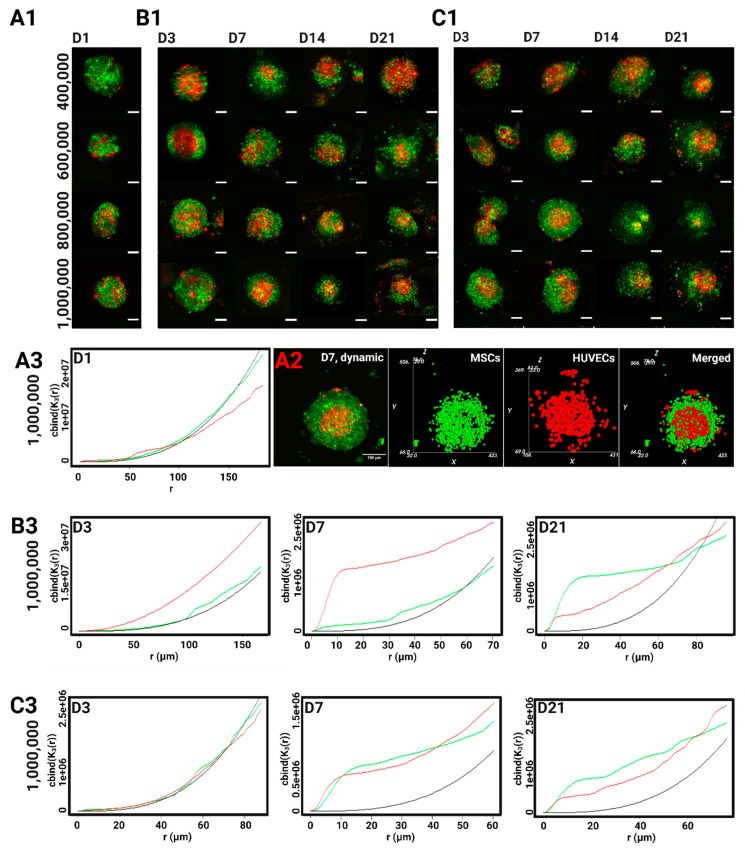
2D representative projections of confocal z-stack acquisitions showing the cellular reorganization of the HUVECs stained with PKH-26 (red) and the MSCs stained with PKH-67 (green) in co-culture spheroids (**A1**) 24-h post-seeding, under (**B1**) static, and (**C1**) the dynamic conditions. Scale bar = 100 µm. (**A2**) the MSCs/the HUVECs spheroid (2D confocal microscopy) after 7 days of dynamic cell culture condition (left). Object-oriented analysis (right). Ripley’s K function describes the spatial distribution of the MSCs (in green) and the HUVECs (in red) with CSD4 (1,000,000 cells/hydrogel scaffold) compared to a random Poisson distribution (in black) (**A3**) 24-h post-seeding, under (**B3**) static cell culture conditions; under (**C3**) dynamic cell culture conditions.

The in situ neovascularization of implanted biological constructs is a lengthy process and involves a myriad of steps. When targeting treatments of large bone defects, the sprouting rate of vessels into the implant site is about 5 µm/h, which is often considered too slow and insufficient to ensure proper perfusion. This emphasizes the importance of implementing pre-vascularization techniques to expedite the integration of the implant into the recipient’s body, enhancing its “pluggability” characteristics [44]. Following the formation of the MSC/HUVEC spheroids 24 h post-seeding onto hydrogels, we wanted to investigate the effects of different culture conditions on the migration and reorganization of the cells. Using confocal microscopy, we analyzed how different 3D culture conditions affect spatial responses across our culture groups by marking the MSCs in green with PKH-67 and the HUVECs in red with PKH-26 prior to seeding (Figure 4(A1,B1,C1)).

The first 24 h post-seeding are crucial for cell aggregation and hence provide the baseline 3D cellular arrangement before inducing any changes to particular environmental conditions. On day 1, the MSCs and the HUVECs seemed to be uniformly mixed within the cell aggregates. By the end of the first week of culture, a definite pattern can be seen across all the culture conditions and densities as the HUVECs migrated inward to form a large cluster predominantly found at the center of the spheroid. It should be clarified that the endothelial cluster was not always isolated at the core as it was not consistently surrounded by mesenchymal aggregates in every direction. In fact, plenty of endothelial cells could still be found at the peripheries and in all directions. This trend persisted during the second and third weeks of culture.

#### 2.4.2. The Detection of Clustering Patterns Across the Entire Diameter of the Spheroid

The spatial reorganization of each cell type was then computationally assessed against a uniform Poisson distribution (Figure 4(A3,B3,C3)). Conforming to confocal observations, the MSCs and the HUVECs behaved spatially very similarly to a Poisson distribution in all the CSDs on day 1. This uniform behavior was more firmly maintained on day 3 in the dynamic conditions, while some endothelial clustering can be observed in the static group as the red curve starts diverging away from the Poisson curve. For the HUVECs, the main clustering event took place on day 7, as its spatial distribution curve significantly concaves above the Poisson curve. In the static culture, this clustering persisted across all the radiuses, i.e., throughout all of the spheroidal volume. On day 21, the HUVECs became dispersed at larger distances, as the red curve dips under the Poisson curve, which corroborates the loss of edge-definition seen in the confocal acquisitions after 2 weeks of culture.

As for the MSCs, the highest incidents of clustering events can be observed on day 7 under the dynamic culture when compared to their uniform arrangement in the static group. Their spatial behavior became extremely similar to that of the HUVECs in the last 2 weeks of the culture, regardless of perfusion, suggesting a possible separation of the cell types, with the spheroids displaying an endothelial center and a more mesenchymal periphery.

Endothelial clustering has been previously described in literature, as seen in the work of Dissanayaka et al. (2014) where they confocally showed a bundle of endothelial clusters distributed throughout the DPSCs/the HUVECs spheroids [45]. Saleh et al. (2011) also described some early central endothelial foci, with the HUVECs being mostly at the periphery, but CD31 staining became increasingly more concentrated at the center of the spheroid after 14 days of culture [46]. Likewise, Marshall et al. (2018) reported the formation of such endothelial foci in the MSCs/ECs spheroids, leading to a partitioning of the two cell types. Interestingly, the authors showed that the inhibition of PDGFRi and ILKi resulted in stronger ECs clustered networks, while the inhibition of FGFRi gave rise to a central endothelial core as seen in many of our spheroids [47]. These pathways might be implicated in the MSC-mediated endothelial rearrangement. On the other hand, other groups have reported a more peripheral or uniform localization of endothelial cells [48,49,50], but their observations were limited to only the first week of culture. Additionally, some publications described vessel-like emergence after 7 days of culture, prompting us to interpret the aggregation of the HUVECs as a possible spatial manifestation of angiogenic events [44,49,51]. Surely, the formation of vessels requires endothelial cells to migrate towards each other before assembling into networks.

Central to this spatial interplay is the composition and mechanical properties of the scaffolds, such as stiffness and viscoelasticity [42]. Additionally, the chemical gradients of growth factors and signaling molecules have a strong effect on cell migration, while cell-cell interactions influence collective migration and multicellular structure formation [52]. Certain spatial formations can optimize these intercellular interactions, be it through direct contact or gap junctions, and consequently induce further spatial evolution. In the case of endothelial cells and osteogenic cells co-culture, the authors reported the upregulation of several transcription factors and molecules directly implicated in bone formation (i.e., Cx43, N-cadherin, and ALP) and vasculogenesis (i.e., VE-cadherin, claudin-1, and occludin-1) [9]. This exchange becomes more relevant for paracrine signaling at higher seeding densities as it increases the number of spheroids and reduces inter-spheroidal distance. The distance was evaluated by Kim et al. (2022) on ASC/HUVEC spheroids, and they observed that a shorter range of around 200 µm between spheroids producing and receiving paracrine signals allowed for optimized diffusion to regulate cell proliferation, endothelial sprouting, and ECM homeostasis [53]. Some of these signals include growth factor families that are directly involved in osteogenic differentiation and tubulogenesis, such as VEGFs, EGFs, and BMPs [54]. Moreover, 3D cultures that produce spheroids of smaller diameters (<200 µm) can improve the production and diffusion of angiogenic molecules (IL-6, ANG, etc.) compared to larger spheroids where necrotic cores are more frequently found and can cause an increase in cellular senescence [55]. The uniform distribution of small-sized spheroids in our hydrogels can therefore help generate similar dimensional conditions for intercellular communication, which can explain the homogeneous spatial behavior seen across different regions of the donut-shaped hydrogel.

### 2.5. Computation of the WSS Within the Bioreactor and Oxygen Concentration Mapping in the Hydrogel

#### 2.5.1. Hydrodynamics

**Figure 5:**
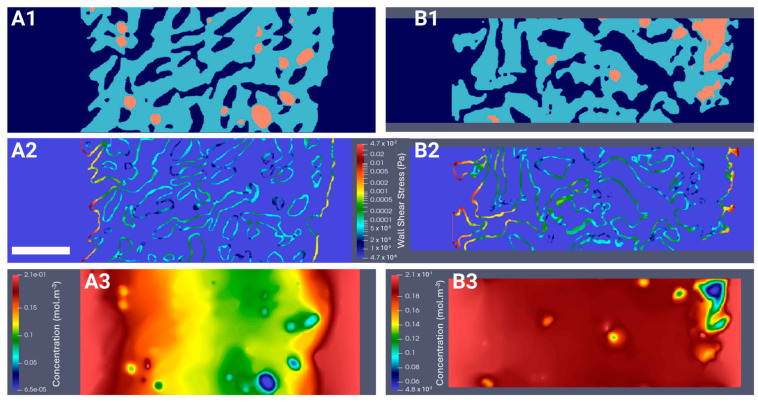
Digital reconstruction of the hydrogel’s microgeometry (in light blue) and the seeded spheroids (in orange) under perfusion flow (in dark blue) for fluid flow simulations with a cell seeding density of 1,000,000 cells (CSD4) on day 1 (**A1**) and day 21 (**B1**). LBM simulations of (**A2**,**B2**) the wall shear stress map (Pa) and (**A3**,**B3**) the dissolved oxygen concentration map (mol.m^−3^) on day 1 and day 21. Scale bar = 500 µm for all images in this figure.

To better understand the biomechanical environment experienced by spheroids in a perfusion bioreactor, we computed the WSS. Indeed, the force exerted by fluid flow on the cell surface can significantly influence cell behavior, including proliferation, differentiation, and alignment [56]. Segments of hydrogels seeded with the highest and lowest cell densities, CSD1 and CSD4, and sampled on day 1 and day 21 were digitally reconstructed (Figure 5(A1,A2) and Figure S2(A1,A2)). These densities were selected to represent the least and most populated scenarios of hydrogel scaffolds. The perfusion flow was simulated in 3D using LBM. The WSS applied by the fluid to the pore walls, the inner and outer edges of the scaffold, and the internal surface of the bioreactor were measured. The average WSS values on the peripheries of the hydrogel varied between 0.19 mPa on day 1 and 0.28 mPa on day 21 for CSD1 (S2(A2, B2)) and between 0.3 mPa on day 1 and 0.19 mPa on day 21 for CSD4 (Figure 5(A2,B2)). At the pore level, the average WSS values remained inferior to 1 mPa. These values are physiologically negligible compared to those reported in the literature and could be insufficient to induce any significant behavioral changes in the cells at any point in the hydrogel [57,58,59].

#### 2.5.2. Oxygen Diffusion

We investigated oxygen transport as a possible factor driving the differences between the spheroids cultured in the static or the dynamic conditions. Using 3D reconstructed segments of seeded hydrogels (Figure 5(A1,B1)), oxygen transport was simulated at the beginning and the end of the culture for the lowest and highest densities. At the scaffold level, the dissolved oxygen concentration was 2.1-fold higher in regions closer to the periphery than to the center (Figure 5(A3,B3) and Figure S2(A3,B3)), coinciding with regions of higher cell proliferation. More specifically, at the spheroid level, the decrease in dissolved oxygen concentration between the outermost and innermost cell layers could reach 98%. Approximately only 0.01% to 5.40% of cells within the hydrogel were subjected to hypoxic conditions, defined as *c* < *K*_*M*_ = 3.8 × 10^−3^ mol.m^−3^. These hypoxic conditions primarily affected the cells located in the spheroid core, which was predominantly endothelial, with the extent of hypoxia, or oxygen transport in general, varying based on the spheroid’s size and location and the density of the neighboring spheroids. The spatial response described earlier can be a unique feature of the HUVEC CRL 1730 line within the bounds of our experimental design. Cell lines may possibly possess different thresholds of the oxygen gradient, and many other factors, compared to primary cells, which are better conditioned to tolerate harsher hypoxic conditions than those calculated in this study.

The evidence in previous research strongly suggests that the spatial distribution of cells is intricately linked to oxygen gradients, with endothelial cells displaying a strong affinity to hypoxic regions [60]. This might stem from an adaptive and compensatory mechanism guiding vessel-sprouting towards hypoxic regions to supply them with oxygen. In fact, oxygen gradients are effective angiogenic triggers even under normoxic conditions, but ultimately induce stronger results when combined with hypoxia [61]. Oxygen gradients can upregulate certain cytokines, such as P1GF, HIF-1α, and VEGF-D, that play an essential role in the migration of endothelial cells, which can ultimately lead to an angiogenic profile [62,63]. However, like most cells, prolonged exposure to hypoxia in vitro can negatively affect endothelial and mesenchymal cell cultures as it increases apoptosis, disrupted ECM remodeling, mitochondrial dysfunction, and cellular senescence [64,65,66,67,68].

Moreover, for CSD4, the fraction of cells in hypoxia was higher on day 1 compared to day 21, despite a 2.0-fold increase in cell number during culture, as the newer cells were primarily located in the more oxygenated edges of the hydrogel. Indeed, dynamic culture allows for adequate oxygen transport even in the most densely populated scaffolds. Since no mechanical stimuli were detected in our dynamic model according to WSS simulations, even in the pores close to the outer and inner borders of the hydrogel where cell proliferation was significantly boosted in dynamic groups, this suggests that the enhanced proliferation induced by perfusion is likely attributed to the optimized mass transport of nutrients and oxygen. After 1 week of culture under the static conditions, the number of cells per scaffold significantly decreased, especially at higher CSDs. The LIVE/DEAD tests did not reveal a decrease in viability until the second and third weeks of culture, which suggested that this cell loss was positively physical in nature. The impaired proliferative functions of cells in static groups did not allow for the compensation of the loss. Further, we cannot demonstrate whether this phenomenon occurred in the scaffolds cultured under the dynamic conditions, as proliferation was highly increased. Alternatively, this may potentially initiate specific adaptive responses within spheroids, leading to a decrease in their packing density to facilitate the diffusion of oxygen and nutrients [69]. Such spatial adjustments can enable the detachment of cells from their 3D aggregates and ultimately from the scaffold entirely.

### 2.6. Osteogenic and Angiogenic Capacities of the MSC/the HUVEC Spheroids

#### 2.6.1. Osteogenic Differentiation of Immortalized MSCs

**Figure 6:**
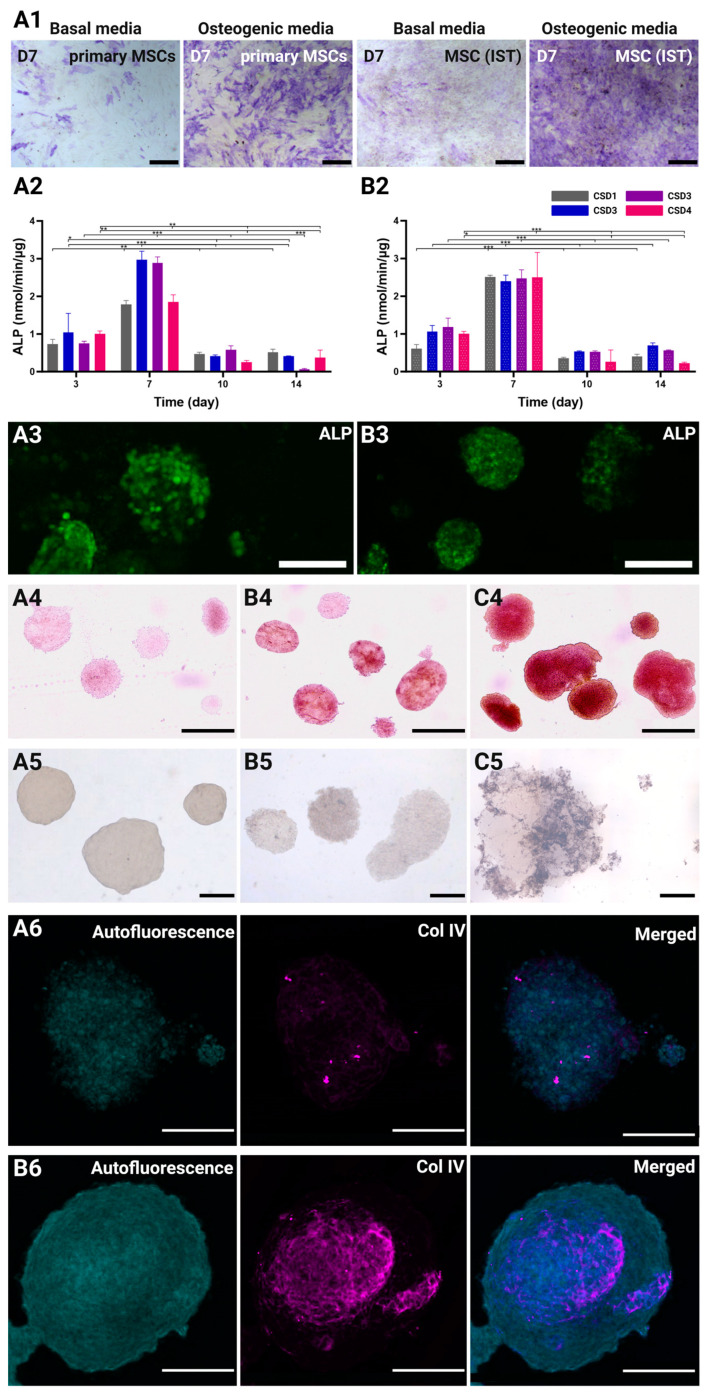
(**A1**) Representative ALP staining test comparing the osteogenic differentiation capacity of the MSC-IST and the primary MSCs on day 7 of the culture. Scale bar = 500 µm. Standardized ALP expression (nmol/min/µg, mean + SEM) throughout the culture period under (**A2**) the static conditions and (**B2**) the dynamic conditions. The cell culture was conducted with CSD1 (400,000 cells/hydrogel scaffold), CSD2 (600,000 cells/hydrogel scaffold), CSD3 (800,000 cells/hydrogel scaffold), and CSD4 (1,000,000 cells/hydrogel scaffold); dynamic culture results are represented with a pattern (░). *, **, and *** denote *p* < 0.05, *p* < 0.01, and *p* < 0.001, respectively. 2D representative projections of confocal z-stack acquisitions showing ALP live stain in co-culture spheroids under (**A3**) static culture conditions and (**B3**) dynamic culture conditions on day 7 with CSD4 (1,000,000 cells/hydrogel scaffold). Scale bar = 100 µm. Alizarin red staining of co-culture spheroids on (**A4**) day 1, and day 21 under (**B4**) the static conditions and (**C4**) the dynamic conditions with CSD4. Scale bar = 300 µm. Von Kossa staining of co-culture spheroids on (**A5**) day 1, (**B5**) day 21 under the static conditions, and (**C5**) the dynamic conditions with CSD4. Scale bar = 100 µm. 2D projections of confocal z-stack acquisitions showing the staining of collagen IV (magenta) in co-culture spheroids under (**A6**) static culture conditions and (**B6**) dynamic culture conditions on day 21 with CSD4. Scale bar = 100 µm.

The MSC-IST, which is how we chose to refer to the MSC line used in our study in this section, and primary MSCs were cultured in differentiation culture media for 7 days, then tested for osteogenic, adipogenic, and chondrogenic markers (Figure 6(A1) and Figure S3). The cells showed a good capacity to respond adequately to differentiation kits, albeit not as strongly as primary MSCs. This is particularly observed in the reduced red oil and alcian blue staining. Nonetheless, the MSC-IST showed very strong ALP staining on day 7, which positively reflected its osteogenic capacity.

#### 2.6.2. Alkaline Phosphatase (ALP) Quantification

Early osteogenic marker ALP was monitored during the first two weeks of culture to assess the effects of different conditions on the MSCs’ differentiation (Figure 6(A2,B2)). The expression of ALP undoubtedly peaked on day 7 for all the culture conditions and CSDs. This results from an average increase of 2.7- and 2.6-fold in the standardized ALP concentration after 1 week of culture in the static and dynamic groups, respectively. The ALP levels then rapidly decreased by 6.0-fold on day 10 and day 14 in all the groups, marking the end of the ALP activity. The control hydrogels prepared in the absence of hydroxyapatite showed little to no ALP expression (Figure S4). This data indicates that day 7 signifies the inception of an osteogenic profile by the MSCs, which also coincides with the spatial clustering of the HUVECs.

While the patterns of standardized ALP expression were highly comparable in all the groups, with no significant effect of the perfusion system on early osteogenesis, CSD2 and CSD3 showed higher peaks by 1.2-fold under the static conditions. On the contrary, CSD1 and CSD4 peaked higher by 1.4-fold under the dynamic conditions. Despite similar ALP expression, the dynamic culture conditions yielded more consistent responses across the different CSDs, possibly due to the uniform culture conditions provided by the perfusion system. In fact, the ALP levels were 2.1-fold higher under the dynamic conditions before standardization by total protein count. The hydrogels cultured under perfusion flow should induce a stronger osteogenic effect at the grafting site the higher the CSD. The ALP live imaging also revealed a uniformly global expression of the ALP protein throughout the spheroid on day 7 under both the static and the dynamic conditions (Figure 6(A3,B3)).

#### 2.6.3. Mineralization of ECM Through Alizarin Red and Von Kossa Staining

Increased mineralization is associated with the late stage of osteogenic differentiation, where the MSCs proliferate, commit to the osteogenic lineage, differentiate into osteoblasts, and produce bone matrix proteins following the upregulation of late osteogenic markers such as osteonectin (ON) and osteopontin (OP) [70,71]. Co-culture spheroids at the end of the culture period were collected and subjected to various staining methods to determine the extent of ECM maturation.

First, the presence of calcium was investigated using Alizarin Red staining on day 21. Compared to the baseline staining seen in spheroids on day 1 (Figure 6(A4)), the spheroids cultured in the static conditions were found to have scarce red patches, indicating the formation of limited zones of mineralization (Figure 6(B4)). Many of the spheroids did not show any positive staining. On the other hand, the staining intensity, coverage, and homogeneity was much greater in the spheroids cultured in the dynamic conditions (Figure 6(C4)).

We also investigated the presence of phosphate deposits using Von Kossa staining. Similarly, the blackish-brown staining was more prominent and widespread in the dynamically cultured spheroids (Figure 6(C5)) than those cultured in the static conditions (Figure 6(B5)). This combined staining approach highlights the enhanced mineralization and ECM maturation achieved under the dynamic culture conditions.

#### 2.6.4. Vasculogenic Capacity of the HUVECs in Co-Culture Spheroids

As pre-vascularization is a critical objective of the 3D MSC/HUVEC co-culture model, it was essential to examine its emerging vasculogenic abilities. Collagen type IV, as a major component of the basement membrane, plays a pivotal role in this process by supporting endothelial cell differentiation, migration, and organization into vascular structures [72]. In our model, collagen type IV is not only directly produced by the HUVECs, but also by the MSCs, assuming their role as pericyte-like cells [73]. Collagen type IV immunocytochemistry revealed a sophisticated mesh-like network of collagen type IV within dynamically cultured spheroids on day 21 (Figure 6(B6)). This network was located at the center of the spheroid, which has been shown to be the hub of endothelial clustering. Conversely, collagen type IV was less dense and underdeveloped in the spheroids cultured in the static conditions (Figure 6(B6)), proving the superior capacity of perfusion systems to promote cell and ECM maturation.

Overall, the continuous flow of culture media in perfusion systems ensures efficient nutrient and oxygen delivery to cells throughout the scaffold, promoting, as seen in our results, cell viability, proliferation, and the enhancement of the osteogenic and angiogenic profiles of the MSC/HUVEC spheroids. Our bioreactor also facilitates the removal of metabolic waste products, maintaining a more stable and favorable microenvironment for cell growth. Generally, perfusion bioreactors enable precise control over flow rates and shear stress, providing the adequate mechanical stimulation of seeded cells, leading to increased osteogenic activity and matrix deposition [74,75,76].

## 3. Conclusions

A biodegradable porous polysaccharide-based hydrogel was produced using a blend of pullulan and dextran crosslinked by sodium trimetaphosphate under saline and alkaline conditions [37,77,78,79]. The architecture of the scaffold consisted of interconnected pores to promote nutrients and oxygen delivery as well as tissue development. The mechanical properties and the bioactivity of the hydrogel were improved with the addition of hydroxyapatite particles for bone tissue engineering applications [31,32,33,80,81]. Strikingly, this hydrogel promotes the formation of spheroids shortly after cell seeding, imparting a simple and succinct three-dimensional cell clustering of high physiological relevance. In our study, we employed a co-culture system containing HUVECs CRL 1730 and SV40+hTERT-immortalized bone marrow-derived MSCs to promote osteogenesis and angiogenesis. The 1:1 co-culture ratio was chosen based on previous findings by Bidarra et al. (2011), which demonstrated enhanced MSCs proliferation and osteogenesis under such conditions [82]. The shift towards 3D cell models is surely justified as they have become valuable tools for studying development, tumor and disease modeling, drug testing, and regenerative medicine applications in general [83]. In particular, spheroids offer complex and heterogeneous architectural structures which better mimic innate physiological interactions and can eventually be used as building blocks to create sophisticated tissues for tissue engineering applications [84].

Our experimental and numerical results strongly suggest that dynamic culture across all cell seeding densities, primarily due to enhanced oxygen and nutrient diffusion, offers significant advantages over static culture, including enhanced cell viability, proliferation, structural integrity in spheroids, and, most importantly, increased osteogenesis and vasculogenesis. This ultimately creates more ideal nucleation buds that serve as foundational points for new bone tissue growth in clinical settings, especially with higher densities.

Future studies may focus on refining perfusion parameters to increase WSS, exploring additional cellular interactions, and translating our findings into clinically relevant applications through in vivo studies. By investing into advanced computational modeling and experimental techniques, we can also continue to explore traditionally inaccessible physiological mapping to help us refine therapeutic procedures with greater precision and biomimicry.

## 4. Materials and Methods

### 4.1. Preparation of Porous Polysaccharide-Based Hydrogel Scaffolds

Porous scaffolds were synthesized according to Grenier et al. (2019) using a polysaccharide blend of pullulan (130 g·L^−1^, Pullulan 200,000, Hayashibara, Japan) and dextran (43.3 g·L^−1^, Dextran 500 kDa Pharmacosmos) crosslinked by sodium trimetaphosphate (STMP, 9.35 10^−2^ mol·L^−1^) under saline conditions (NaCl, 3.46 mol·L^−1^) and alkaline conditions (NaOH, 0.953 mol·L^−1^) [37]. Hydroxyapatite (HAp) was added by replacing 22.97 mL of distilled water by an HAp particles solution (36 mg·L^−1^) in the pullulan/dextran/sodium mixture. After crosslinking, the gel was molded into a 1 mm thick sheet for 20 min at 50 °C, resulting in higher viscosity and a more hardened structure. The sheets were then cut using a stainless-steel punch tool (5.5 mm in diameter) to create round discs. The samples were neutralized in a phosphate buffer (PBS 10x) and distilled water, and were then washed overnight in saline solution (NaCl, 0.250 g·L^−1^) until equilibrium. The swollen hydrogel discs were placed (the base was horizontal) on Petri dishes (VWR, 391-0875) in batches of 50 before undergoing freeze-drying in a MUT freeze-drier (MUT 004, Cryotech^®^, Voujeaucourt, France). The plates were initially kept at 15 °C and the cooling rate was set to −0.1 °C min^−1^ until −20 °C. Freezing occurred after nucleation at –10 ± 2 °C. Post freeze-drying, a central hole (3.5 mm diameter) was cut in the hydrogel discs using a biopsy punch (Dominique Dutscher, Bernolsheim, France). In their dry state, the scaffolds had a diameter of approximately 9 mm and a thickness of 1.6 mm.

### 4.2. X-ray Computed Microtomography Hydrogel Characterization

#### 4.2.1. Image Acquisition

The 3D structure of the freeze-dried hydrogels was obtained using an X50CT X-ray microtomography (NorthStar Imaging, Rogers, AR, USA). The X-ray scans produced grayscale images representing the internal structure, with a voxel size of (8.4 μm × 8.4 μm × 8.4 μm) during a 30 min acquisition period. A microfocus tube (XWT-190-TC), operated at 70 kV and 300 μA, was used. Zooming during a 3 h acquisition achieved a higher resolution of (1 μm × 1 μm × 1 μm), specifically to characterize the sizes of the HAp clusters.

#### 4.2.2. X-ray Computed Microtomography Image Reconstruction, Visualization, and Analysis

Image reconstruction was performed on a server with 4 GPU cards (NVidia RTX Get Force) and took approximately 5 min. Avizo version 2019.1 software (ThermoFisher Scientific France, Asnières-sur-Seine, France) was used for the visualization, analysis, and 3D modeling.

### 4.3. Cell Lines

SV40+hTERT-human immortalized, bone marrow-derived Mesenchymal Stem Cells (GeneCopoeia, Catalog number SL428, Rockville, MD, USA) were used. The human bone marrow MSCs were immortalized via transfection with lentiviruses carrying SV40 and hTERT. The cells were cultured with αMEM (nucleosides, no ascorbic acid) (Gibco by Thermo Fisher Scientific France, Asnières-sur-Seine, France), supplemented by 10% (mL·mL^−1^) bovine fetal serum (Dominique Dutscher, Dubernolsheim, France), and decomplemented at 56 °C for 30 min and by 1% (mL·mL^−1^) penicillin and streptomycin (10,000 Units·mL^−1^–10 mg·mL^−1^) (PAN-Biotech GmbH, Aidenbach, Germany). The cells were expanded in 150 cm^2^ cell culture flasks (Techno Plastic Products AG, TPP, Trasadingen, Switzerland) at a density of 5 × 10^5^ cells.cm^−2^ at 37 °C (95% air, 5% CO_2_). The HUVEC CRL 1730 from ATCC (American Type Culture Collection, ATCC, Manassas, VA, USA) were expanded using IMDM (Gibco by Thermo Fisher Scientific France, Asnières-sur-Seine, France) and supplemented by 20% (mL·mL^−1^) bovine fetal serum, 0.4% endothelial cell growth supplement/heparin (PromoCell, Heidelberg, Germany), and 1% penicillin and streptomycin. The HUVECs were also expanded in 150 cm^2^ culture flasks at a density of 10^6^ cells.cm^−2^ at 37 °C (95% air, 5% CO_2_). To carry out the co-culture experiments under either the static or the dynamic culture conditions, we used a 1:1 mixture of EBM^TM−2^ Endothelial cell growth Basal Medium supplemented with EGM^TM−2^ MV Microvascular Endothelial SingleQuots^TM^ kit (LONZA, Basel, Switzerland) and αMEM (nucleosides, no ascorbic acid) (Gibco by Thermo Fisher Scientific France, Asnières-sur-Seine, France).

#### SV40+hTERT-Immortalized Bone Marrow-Derived Mesenchymal Stem Cells Differentiation Capacity

To check the differentiation capabilities of the SV40+hTERT-immortalized, bone marrow-derived MSCs, the cells were cultured in chondrogenic differentiation medium (StemPRO Medium + Chondrogenesis differentiation kit), adipogenic differentiation medium (StemPRO Medium + Adipogenesis differentiation kit), and in osteogenic differentiation medium, (StemPRO Medium + Osteogenesis differentiation kit). To demonstrate the osteogenic differentiation, after 7 days of culture, an ALP staining test was performed; for adipogenic differentiation, after 14 days of culture, a red oil staining test was performed; for chondrogenic differentiation, after 14 days of culture, an alcian blue staining test was performed. The stainings were observed using an inverted phase contrast microscope.

### 4.4. Cell Seeding Within 3-D Macroporous Scaffold

Prior to cell seeding, dried scaffolds (8.4 mm in diameter, a central hole 3.5 mm in diameter, thickness of 1.5 mm) were sterilized under a UV lamp for 80 min total, 40 min on each side (L-215.G, Bioblock Scientific by ThermoFisher, Asnières-sur-Seine, France). The cell membranes were previously labeled with red dye PKH-26 (the MSCs) or green dye PKH-67 (the HUVECs) (Sigma-Aldrich, St. Louis, MS, USA) according to the manufacturers’ instructions. The cells were detached with Trypsin/EDTA 0.05%/0.02% (PAN-Biotech GmbH, Aidenbach, Germany), washed with complete medium, counted, and centrifuged. The MSCs and the HUVECs were mixed at a 1:1 ratio and cultured for 1, 3, 7, 14, and 21 days at 400,000 (CSD1), 600,000 (CSD2), 800,000 (CSD3), and 1,000,000 (CSD4) cells/hydrogel scaffold for the spheroid distribution and size, the 3D spatial reorganization of the MSCS/the HUVECs, proliferation, and ALP assays.

The seeding protocol requires suspension of the cells in a volume of 40 µL/hydrogel of co-culture media mixture. The cells were seeded on top of the surface of the porous hydrogel scaffolds in 4 directions (10 µL up, 10 µL down, 10 µL right, 10 µL left). The swelling of the scaffold following the seeding of cell suspension, as well as the lack of any adhesion sites, allowed the cells to assemble and aggregate together inside of the pores, compacting due to surface tension and cohesive forces, in a similar fashion to well suspension methods. The total swelling was obtained by gradually adding 80 µL of medium per scaffold. For the 3D static culture, seeded porous hydrogel scaffolds were incubated under standard cell culture conditions in 6-well plates, 4 scaffolds per well, with 4 mL of culture medium refreshed every 3 days. The cell viability and mortality within these hydrogel scaffolds was assessed using the LIVE/DEAD assay (Invitrogen by Thermo Fisher Scientific France, Asnières-sur-Seine, France).

In all the experiments, N = 3 seeded hydrogels and N = 50 spheroids/samples were collected at every timepoint, seeding density, and culture condition. The confocal spheroid acquisitions (Zeiss LSM 700, Rueil Malmaison, France) were done in z-stack mode, over 370 µm depth by 200 XY slices with 2.5 µm resolution (XY is parallel to the scaffold base).

### 4.5. Optical Coherence Tomography Hydrogel Characterization

#### 4.5.1. Image Acquisition

The 3D porous structure of scaffolds in their hydrated state was captured using Optical Coherence Tomography (OCT, Thorlabs Ganymede series 621C1, Newton, MA, USA), equipped with a motorized stage (High-Speed Thorlabs MLS203, Newton, MA, USA). The hydrogels were hydrated using the co-culture medium comprising a 1:1 ratio mixture of EBM^TM−2^ endothelial cell growth basal medium supplemented with an EGM^TM−2^ MV Microvascular Endothelial SingleQuots^TM^ kit (LONZA, Basel, Switzerland) and αMEM (nucleosides, no ascorbic acid) (Gibco by Thermo Fisher Scientific France, Asnières-sur-Seine, France), then placed in an incubator at 37 °C with 95% air and 5% CO₂. The OCT was configured with a refractive index of 1.33 and a nominal wavelength of 900 nm, operating at an acquisition frequency of 10 kHz (A-scan). 3D images were obtained with an axial Z resolution of 1.45 μm over a total depth of 1 mm during a 20 min acquisition period. The spatial resolution in the XY plane was (8 μm × 8 μm).

#### 4.5.2. Image Reconstruction, Visualization, and Analysis

OCT acquisitions were converted to 8-bit files before being processed with Avizo software (ThermoFisher Scientific). The pore space within was segmented in 3D using Avizo software. The segmentation process involved Gaussian filtering, thresholding, noise elimination (despeckle), object separation (watershed algorithm), and labeling. Pore size was defined as the cubic root of the 3D pore volume. When the hydrogel scaffolds were seeded with cells, the spheroids were manually colored using Avizo to facilitate their further segmentation. Finally, the images were resampled to adjust the voxel dimensions to a cubic size of 8 μm.

### 4.6. Dynamic Culture in a Perfusion Bioreactor

**Figure 7:**
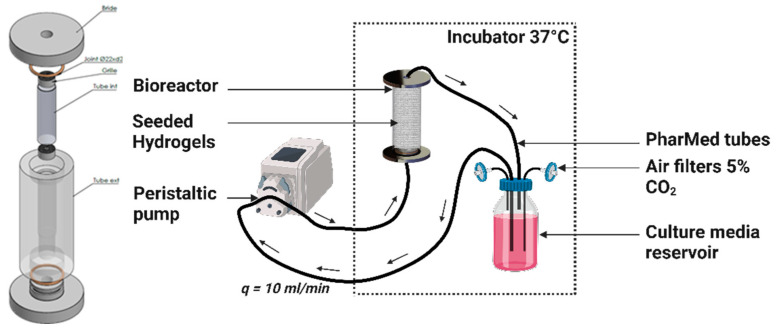
Custom made perfusion bioreactor and bioreactor circuit containing centrally aligned seeded hydrogels. Perfusion flow rate q = 10 mL·min^−1^.

At 24 h post-seeding, the seeded porous hydrogel scaffolds were vertically stacked over approximately 3 cm height inside a polypropylene (PP) tube (12 mm inner diameter and 4 cm height) and held between two Teflon grids (5 mm height) with 100 holes of 1.2 mm in diameter and 50% porosity (Figure 7).

The annular channel between the inner wall of the PP tube and the periphery of the hydrogel scaffold, as well as the circular channel formed by stacking centrally holed discs, facilitated the flow of the culture medium. The PP tube was transferred to the bioreactor, which consisted of a cylindrical chamber made of polycarbonate (PC) that wasconnected to an external culture media reservoir through flexible silicon tubes of 3.2 mm inner diameter (R6504—26BPT, PharMed, Saint-Gobain Tuyaux, Pont-à-Mousson, France). The reservoir was filled with 100 mL of culture medium, which was refreshed every 3 days, equilibrated at pH = 7.2 with CO_2_-enriched air (5%) at a 10 mL·min^−1^ air flow rate. The silicon tubes were hooked to a peristaltic pump (Easy-load 3, Masterflex, Cole-Parmer, Chicago, IL, USA) allowing a perfusion rate or volumetric flow rate of q = 10 mL·min^−1^. The superficial velocity was calculated to be $v_{s}=$ 1.47 mm·s^−1^ through the empty PP tube cross section. The bioreactor circuit was interrupted at multiple timepoints to collect hydrogel samples under completely sterile conditions by transferring the circuit under the cell culture hood and using sterile surgical tweezers.

### 4.7. The MSC and HUVEC Coculture

#### 4.7.1. Cell Proliferation Assay

Cell proliferation in different culture conditions was measured using the CyQUANT™ Cell Proliferation Assay (Invitrogen by Thermo Fisher Scientific France, Asnières-sur-Seine, France). At every timepoint, 4 hydrogels were collected from each group then individually digested using an enzymatic solution containing 10% microbial pullulanase (Sigma-Aldrich, Heidelberg, Germany) and 5% dextranase (Sigma-Aldrich, Heidelberg, Germany) in PBS. The cells were then centrifuged (5 min, 300 RCF) and washed 2 times in PBS, then frozen at −20 °C. On the day of the experiment, each sample of cells was brought to room temperature then suspended in 600 µL of the CyQUANT^®^ GR dye/cell-lysis buffer solution, which was prepared according to the manufacturer’s instructions to generate triplicates in 96-well plates. The plates were measured in the Multiskan GO microplate reader (Thermo Fisher Scientific France, Asnières-sur-Seine, France).

#### 4.7.2. Cell Viability Assessment

A LIVE/DEAD kit (Invitrogen by Thermo Fisher Scientific France, Asnières-sur-Seine, France) was used to evaluate the viability and the mortality of the cells. The seeded hydrogel scaffolds were washed 1× with a PBS, then incubated in 500 µL of PBS containing calcein-AM (1 µL 8 mM) and ethidium homodimer-1 (2 µL 4 µM) for 90 min on a plate shaker at room temperature. The hydrogels were then analyzed immediately under the CLSM with 1.25 µm resolution. Selected spheroids were observed over 370 µm depth with 200 XY slices at 2.5 µm resolution.

#### 4.7.3. Alkaline Phosphatase (ALP) Activity Assay

ALP was measured as an early osteoblastic marker using the colorimetric ALP Assay Kit (Abcam, Paris, France). The kit uses p-nitrophenyl phosphate (pNPP) as a phosphatase substrate, which turns yellow (max = 405 nm) when dephosphorylated by ALP. The seeded hydrogels were first digested using an enzymatic solution containing 10% microbial pullulanase (Sigma-Aldrich, Heidelberg, Germany) and 5% dextranase (Sigma-Aldrich, Heidelberg, Germany) in a PBS. The collected cells were then washed twice with the PBS, then suspended in 500 µL of ALP assay buffer to undergo cell lysis (Precellys Evolution Touch Homogenizer, Bertin Technologies, Montigny-le-bretonneux, France). The standard and sample wells were then processed according to the manufacturer’s instructions. After stopping the reaction, the plates were placed in the Multiskan GO microplate reader (Thermo Fisher Scientific France, Asnières-sur-Seine, France) at 405 nm. The enzyme activity (expressed as units/g of total protein) was normalized by total protein concentration, detected by a BCA protein assay kit (Thermo Fisher Scientific France, Asnières-sur-Seine, France).

#### 4.7.4. ALP Live Stain

ALP was visualized in live samples using the ALP Live Stain (Invitrogen by Thermo Fisher Scientific France, Asnières-sur-Seine, France). The seeded hydrogels were collected from their respective culture conditions, washed 3× with serum-free media, then incubated in 1 mL of αMEM (nucleosides, no ascorbic acid) (Gibco by Thermo Fisher Scientific France, Asnières-sur-Seine, France) containing 2 µL of ALP Live Stain (500x) for 30 min. The samples were washed twice with serum-free media, then the spheroids were quickly observed on the confocal microscope (ZEISS LSM 700, Rueil Malmaison, France).

#### 4.7.5. Mineralization

1. Von Kossa staining

The Von Kossa staining method specifically identifies phosphate ions by reacting with silver nitrate, forming black or brownish deposits where phosphate is present. A Silver Plating Kit acc. to Von Kossa (Sigma-Aldrich, Heidelberg, Germany) was used on the seeded hydrogels fixed in 4% PFA. The samples were incubated with silver nitrate solution for 30 min under a UV lamp, and then with sodium thiosulfate solution for 10 min. The hydrogels were washed between the steps under running tap water for at least 5 min. The spheroids were isolated using an enzymatic solution containing 10% microbial pullulanase (Sigma-Aldrich, Heidelberg, Germany) and 5% dextranase (Sigma-Aldrich, Heidelberg, Germany) in PBS, then washed 5× with PBS. Observations were made on optical microscopy (ZEISS, Axioscope 7, Rueil Malmaison, France).

2. Alizarin red staining

Matrix mineralization was further evaluated using the Alizarin-Red Staining Solution (Sigma Aldrich) to detect calcium deposits, a key indicator of bone formation. The staining solution was diluted with equal amount of distilled water then added to samples fixed in 4% PFA for a 30 min incubation period. The hydrogels were washed between the steps under running tap water for at least 5 min. The spheroids were isolated then washed 5× with PBS. Observations were made on optical microscopy (ZEISS, Axioscope 7, Rueil Malmaison, France).

3. Collagen type IV immunocytochemistry

Vasculogenesis in the HUVECs was assessed by monitoring the development of collagen type IV, which is an essential component of the ECM that supports and stabilizes the formation of new blood vessels. The spheroids were fixed then incubated for 48 h with Rabbit anti-collagen IV primary antibody (ab6586, Abcam) (1:100 in PBS) on a plate shaker at 4 °C. Each sample was washed repeatedly in a PBS over the course of 48 h before adding the secondary Goat anti-Rabbit antibody (A-21244, Invitrogen by Thermo Fisher Scientific France, Asnières-sur-Seine, France). Washing steps were repeated over the course of 48 h before undergoing confocal observations (ZEISS LSM 700, Rueil Malmaison, France).

### 4.8. Confocal Image Reconstruction, Visualization, and Spatial Analysis

Z-stack images were initially analyzed in ImageJ 2.14.0/1.54f to create 2D Z projections and do initial 3D evaluations using 3D Viewer and 3DSuite plugins.

#### 4.8.1. 3D Plots

As 3D clustering coupled with our selected staining method renders it extremely difficult to visualize the borders of adjacent cells, classical cell segmentation algorithms were not applicable. In order to move from a pixel-oriented to an object-oriented analysis, the red and green channels were first isolated and treated separately, which would pertain to the MSCs’ and the HUVECs’ analysis, respectively.

Using python libraries (scikit-image, mayavi, pandas), a 3D maxima finder algorithm was applied to each channel to identify the coordinates of cell-representing fluorescent clusters. Next, 3D spherical objects were plotted around the extracted coordinates to create objects of appropriate cell sizes. For the immortalized lines used in this article, we estimated the average size of the MSCs to be 15 µm × 15 µm × 15 µm and the average size of the HUVECs to be 7 µm × 7 µm × 7 µm.

#### 4.8.2. Ripley’s K Function

Ripley’s K function measures the spatial dependence or clustering of events by comparing the observed distribution of events to a theoretical random distribution within a defined geographic area, such as the bounds of a spheroid. Using R libraries (spatstat, spdep, readxl), we observed the spatial distribution of the MSCs and the HUVECs independently using the K3est function.

(1) $$K_{3}\left(r\right)=\frac{1}{\lambda }\;Ε\;\left(Ν\left(\Phi ,\;x,\;r\right)\;|\;x\in \Phi \right)$$

According to the documentation in R, λ is the expected number of points/events per unit volume, and $Ν\left(\Phi ,\;x,\;r\right)$ is the number of points of Φ, representing the coordinates of cells of belonging to a certain type (the MSCs or the HUVECs), within distance r of point x, representing the spatial coordinates of the current cell being examined.

By calculating Ripley’s K function for various values of r, we created a K(r) plot against a Poisson distribution. When the observed K value is larger than the Poisson distribution for a particular distance, the distribution is more clustered than a random distribution at that distance (the scale of analysis). When the observed K value is smaller than the Poisson distribution, the distribution is more dispersed than a random distribution at that distance.

Given that we are dealing with replicated point patterns as we collect and observe multiple samples, we used the pooling function to average the K functions of all the replicates, as described in chapter 16, section 8 of Baddeley et al.’s book [85].

### 4.9. Computational Fluid Dynamics (CFD) Simulation of Porous Scaffold Perfusion

#### 4.9.1. Modeling and Lattice Boltzmann Method (LBM) Implementation

The culture medium was assumed to be incompressible, characterized by a fluid density ρ=993 kg·m^−3^, and Newtonian, with constant dynamic viscosity $\mu =10^{-3}$ Pa·s at 37 °C [86]. The fluid dynamics are governed by the continuity equation and the Navier–Stokes momentum equations. These equations describe the fluid flow around the hydrogel scaffolds stacked in the bioreactor, as well as in the pores of the hydrogel scaffolds. The bioreactor walls, the hydrogel domains (within the porous hydrogel scaffolds), and the cellular structures, were treated as impermeable solids, implying that the fluid velocity at the fluid–solid interface was zero. The two-relaxation-time Lattice Boltzmann Method (TRT-LBM), employing a three-dimensional cubic lattice with nineteen discrete velocities (D3Q19), was utilized to simulate the Navier–Stokes equations [87]. A detailed implementation of the method was described by Duval et al. (2006) and by Thibeaux et al. (2019) [88,89]. The following provides a summary of the general aspects of the method and an introduction to the underlying parameters.

In this LBM, the fluid is modeled using a population of fictitious particles characterized by a discrete velocity distribution of Q=19 vectors. These particles are constrained to move along the 3D lattice. A two-step process governs the particle dynamics at each time step. First, the particles propagate according to their velocity from one lattice node to a neighboring one. Second, the particle velocity distribution undergoes relaxation towards the equilibrium distribution. The fluid velocity is derived from the first-order moment of the particle velocity distribution.

The relaxation involves two parameters. First, $s_{1}$ is directly related to the kinematic viscosity of the real fluid ν=μ/ρ by

(2) $$s_{1}={\left(\frac{\nu }{c_{s}^{2}}\frac{∆t}{{∆x}^{2}}+\frac{1}{2}\right)}^{-1}$$

where $c_{s}$ is the intrinsic speed of sound of the LBM ($c_{s}^{2}=1/3$ in lattice units), ∆x is the lattice spacing (in real units), and ∆t is the time step (in real units). Second, $s_{2}$ is computed from $s_{1}$ with the following so-called “magic number relation” [90]:

(3) $$s_{2\;}=8\frac{2{-s}_{1}}{8-s_{1}}$$

The no-slip condition at the solid boundaries was modeled using bounce-back reflection. Since the two relaxation parameters satisfy Equation (3), the solid walls are located halfway between a fluid node and a solid node [90]. Finally, the time step is selected to keep the effects of LBM intrinsic compressibility negligible:

(4) $$∆t=0.06∆x/v_{\max}$$

where $v_{\max}$ is the maximal fluid velocity. $v_{\max}$ is reached on the symmetry axis of the circular channel formed by the stacked perforated disks. Assuming Poiseuille flow in the circular channel, the maximal fluid velocity was estimated at $v_{\max}=10\;\mathrm{mm}\cdot s^{-1}$.

To determine the wall shear stress (WSS) that the fluid exerts, the viscous stress tensor $\overset{̿}{\sigma }$ and the wall normal vector $\vec{n}$ are required. The modulus of WSS τ is given by

(5) $$\tau =\left|\overset{̿}{\sigma }\cdot \vec{n}-\left(\left(\overset{̿}{\sigma }\cdot \vec{n}\right)\cdot \vec{n}\right)\vec{n}\right|$$

The viscous stress tensor was calculated at each neighbor node of a solid surface (hydrogel or bioreactor lateral inner walls) from the LBM velocity distribution function at that node [89,91]. The determination of the boundary normal vector was not as straightforward, due to the staircase shape of the solid boundary. We implemented a technique proposed by Matyka et al. (2013) to detect the normal wall direction locally [92]. This method relies on the geometry of the solid boundary.

#### 4.9.2. Computational Configuration

In order to reduce the computational time, fluid flow simulations were performed on a parallelepipedal segment, representative of the bioreactor (the curvature of the real geometry was neglected). This segment consisted of three parts associated with the circular inner channel, the porous hydrogel scaffolds, and the annular outer channel, respectively. The microgeometry of the porous hydrogel and the seeded spheroids was extracted from reconstructed 3D images acquired by OCT. We tested 3D images of scaffolds with CSD1 or CSD4 and sampled after 1 day or 21 days of the dynamic culture within a perfusion bioreactor.

**Figure 8:**
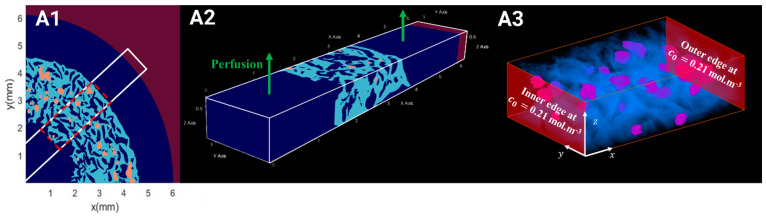
(**A1**) Reconstruction of OCT acquisition representing the hydrogel’s microgeometry (in light blue) and the seeded spheroids (in orange) under perfusion flow (in dark blue) for fluid flow simulations. (**A2**) Selected ROI (6400 μm × 1200 μm × 640 μm) for hydrodynamics simulations. (**A3**) Selected ROI (2576 μm × 1200 μm × 640 μm) of hydrogel (in blue) with seeded spheroids (in purple) for oxygen transport.

The computational domain dimensions were 6.4 mm length in the X-direction, 1.2 mm width in the Y-direction, and 0.64 mm height in the Z-direction parallel to the bioreactor axis (Figure 8). The height of the domain corresponded to the half-thickness of a hydrogel scaffold. A symmetry boundary condition was set on the plane X=0 mm. No-slip condition was set on the plane X=6.4 mm. Periodic boundary conditions were set in the Y and Z directions mimicking a regular stack of scaffolds. The lattice spacing was ∆X = 8 μm. The time step ∆t satisfied Equation (4). The perfusion flow within the bioreactor segment was generated by a body force. The body force was adjusted such that the fluid velocity in the plane X=0 mm matched the estimated fluid velocity along the bioreactor axis, assuming Poiseuille flow. Then, about 1,000,000 time steps were required to reach a steady state. The results were exported and visualized using ParaView version 5.11.2 software.

### 4.10. Simulation of Oxygen Transport and Consumption Within a Porous Scaffold

#### 4.10.1. Modeling and LBM Implementation

Oxygen transport within the scaffolds was described by the convection–diffusion equation:

(6) $$\frac{\partial c}{\partial t}+\vec{v}\cdot \vec{\nabla }c=∆c+r_{O_{2}}^{\alpha }$$

where c (mol·m^−3^) is the local dissolved oxygen concentration, and α refers to the domain where dissolved oxygen is transported. The fluid domain is denoted “0”, the hydrogel domain as “hyd”, and the spheroid domain as “sph”. $\vec{v}$ is the hydrodynamic velocity equal to zero everywhere except in the pores of the scaffold (i.e., fluid domain). Given the high dilution of dissolved oxygen in the culture medium, the diffusion flux was modelled by Fick’s law with diffusion coefficient $D_{O_{2}}^{\alpha }$. $r_{O_{2}}^{\alpha }$ represents the (algebraic) oxygen production rate per unit volume. $r_{O_{2}}^{\alpha }=0$ in the fluid and hydrogel domains. In the spheroid domain, $r_{O_{2}}^{\alpha }$ is associated with the cellular (MSCs and HUVECs) consumption of oxygen and was modeled using Michaelis–Menten-like kinetics:

(7) $$r_{O_{2}}^{\mathrm{sph}}=-\rho_{\mathrm{MSC}}V_{\max}^{\mathrm{MSC}}\frac{c}{c+\;K_{M}^{\mathrm{MSC}}}-\rho_{\mathrm{HUVEC}}V_{\max}^{\mathrm{HUVEC}}\frac{c}{c+K_{M}^{\mathrm{HUVEC}}}$$

where $\rho_{\mathrm{MSC}}$ (resp. $\rho_{\mathrm{HUVEC}}$) is the MSC (resp. HUVEC) density within the spheroids, $V_{\max}^{\mathrm{MSC}}$ (resp. $V_{\max}^{\mathrm{HUVEC}}$) is the maximal oxygen consumption rate of the the MSCs (resp. HUVECs), and $K_{M}^{\mathrm{MSC}}$ (resp. $K_{M}^{\mathrm{HUVEC}}$) is the Michaelis–Menten-like constant of the MSCs (resp. HUVECs), that is to say, the oxygen concentration where oxygen consumption is reduced by 50%. $\rho_{\mathrm{MSC}}$ and $\rho_{\mathrm{HUVEC}}$ were experimentally estimated at $3.54\times 10^{14}$ m^−3^. The MSC kinetic constants were set to $V_{\max}^{\mathrm{MSC}}=5.4\times 10^{-17}$·mol·cell^−1^·s^−1^ and $K_{M}^{\mathrm{MSC}}=3.8\times 10^{-3}$·mol·m^−3^, the HUVEC constants to $V_{\max}^{\mathrm{HUVEC}}=2.4\times 10^{-17}$·mol·cell^−1^·s^−1^ and $K_{M}^{\mathrm{HUVEC}}=5\times 10^{-4}$·mol·m^−3^ [93,94].

In the fluid phase, the dissolved oxygen diffusion coefficient was $D_{O_{2}}^{0}=3.3\times 10^{-9}$ m^2^·s^−1^, assimilating the culture medium to pure water at 37 °C. In the hydrogel phase, the oxygen diffusion coefficient was measured by Grenier et al. (2023), i.e., $D_{O_{2}}^{\mathrm{Hyd}}=1.6\times 10^{-9}$ m^2^·s^−1^ [30]. In the spheroids, the oxygen diffusion coefficient was assumed to be $D_{O_{2}}^{\mathrm{sph}}=3\times 10^{-10}$ m^2^·s^−1^ [95]. The oxygen partition coefficients between the fluid and the hydrogel, and between the hydrogel and the cells, were assumed to equal 1.

A TRT-LBM with a cubic lattice in three dimensions and 7 velocities (D3Q7) were used to simulate the convection–diffusion equation as proposed by Ginzburg (2017) [96]. The dissolved oxygen was represented by a population of fictitious particles, distinct from that representing the fluid. The dissolved oxygen concentration is given by the zeroth order moment of the particle velocity distribution.

The relaxation of the particle velocity distribution depended on two parameters, $s^{-}$ and $s^{+}$. $s^{-}$ is related to the oxygen diffusion coefficient in the considered phase. In the hydrogel, $s^{-}$ is given by

(8) $$s^{-}={\left(\frac{D_{O_{2}}^{\mathrm{Hyd}}∆t}{c_{e}{∆x}^{2}}+\frac{1}{2}\right)}^{-1}$$

where $c_{e}$ is a scale parameter fixed at $c_{e}=1/4$. The relaxation parameter $s^{+}$ satisfies the “magic number relation”, which presently expresses as $s^{+}=2-s^{-}$ [96]. The time step was chosen to ensure the stability of the D3Q7 LB method, which implies

(9) $$∆t\;\leq \frac{∆x}{2v_{\max}}\;$$

where $v_{\max}$ is the maximal velocity reached by the fluid in computational domain. Simulations were run until the dissolved oxygen concentration field reached a steady state. Results were exported and visualized using ParaView version 5.11.2 software.

#### 4.10.2. Computational Configuration

We introduced $c_{0}$, the concentration of dissolved oxygen when the culture medium was in equilibrium with air at 1 atm. We considered that the culture medium entered the bioreactor with the concentration $c_{0\;}=0.21$ mol·m^−3^, corresponding to the oxygen equilibrium concentration in pure water at 37 °C. In the bioreactor, the transport of dissolved oxygen from the culture medium to the cells is a three-step process, i.e., (i) the convective transport in the circular and annular channels, (ii) the convecto-diffusive transport in the porous hydrogel, and (iii) the diffusional transport and consumption within the spheroids. We demonstrated in a precedent paper that the convective transport in the channels is not limitative [30]. Furthermore, the consumption rate by the spheroids represents, at the most, 5% of the oxygen molar flow rate entering the bioreactor. Therefore, we can reasonably assume that the oxygen concentration of the liquid phase in the circular and annular channels is approximately homogeneous and equal to $c_{0}$.

It is relevant to estimate the Thiele modulus (ϕ) that accounts for the competition between the kinetics of oxygen consumption by the cells and the limitation of oxygen transport by diffusion. For a spheroid of radius $r_{\mathrm{sph}}$, the Thiele modulus reads

(10) $$\phi =\frac{\left(\rho_{\mathrm{the}\;\mathrm{MSC}}V_{\max}^{\mathrm{the}\;\mathrm{MSC}\;}+\rho_{\mathrm{the}\;\mathrm{HUVEC}}V_{\max}^{\mathrm{the}\;\mathrm{HUVEC}}\right)r_{\mathrm{sph}}^{2}}{D_{O_{2}}^{\mathrm{sph}}c_{0}}$$

assuming that the oxygen concentration around the spheroid is of the order of $c_{0}$. For $r_{\mathrm{sph}}=50$ μm, we find ϕ≅1, corresponding to the intermediate regime between the “reaction-controlled” regime (ϕ≪ 1) and the “diffusion-controlled” regime (ϕ≫ 1).

As compared to the computational domain used for the CFD simulations, the domain for the oxygen transport simulations can be reduced to its scaffold part, i.e., a parallelepipedal segment of 2.6 mm length in the X-direction, 1.2 mm width in the Y-direction, and 0.64 mm in the Z-direction. In this new frame of reference, the dissolved oxygen concentration was set at $c_{0}$ on the planes X=0 mm and X=2.6 mm. Periodic conditions were set in Y and Z directions to mimic a regular stack of scaffolds. The lattice spacing was identical to the one used for the CFD simulations, i.e., ∆X = 8 μm, and we used the same 3D images of scaffolds seeded either with CSD1 or CSD4 and sampled after 1 day or 21 days of the dynamic culture within a perfusion bioreactor. The time step ∆t satisfied Equation (9). About 700,000 time steps were required to reach a steady state.

### 4.11. Data Analysis

All the experiments were performed at least in triplicate. At least 50 spheroids were closely inspected per sample, when relevant. The results are presented as mean ± SEM. Two-way repeated measures ANOVA with Tukey post hoc tests were performed on IBM SPSS Statistics version 29.0.1.0 software. The statistical figures were made on Graphpad Prism version 10.0.1 software. Statistical significance was denoted as * *p* < 0.05; ** *p* < 0.01; and *** *p* < 0.001.

## Figures and Tables

**Table 1:** Comparison of the average volume and total number of formed spheroids per hydrogel with respect to the initial cell seeding density at 24 h post-seeding.

	CSD1	CSD2	CSD3	CSD4
Cell Seeding Density (CSD)/scaffold	400,000	600,000	800,000	1,000,000
Average spheroid volume (µm^3^) × 10^5^	6.0 ± 0.8	7.6 ± 0.1	6.3 ± 0.4	10.9 ± 0.1
Spheroid number	773 ± 252	805 ± 54	2384 ± 203	1368 ± 249

## Data Availability

The original contributions presented in the study are included in the article/Supplementary Materials, further inquiries can be directed to the corresponding author.

## Supplementary Materials

The following supporting information can be downloaded at: https://www.mdpi.com/article/10.3390/gels10100666/s1, Figure S1: White field acquisitions of seeded hydrogel sections showing the distribution of MSCS/HUVECs spheroids in (A) static culture conditions and (B) dynamic culture conditions in a perfusion bioreactor. Cell culture was conducted with CSD1 (400,000 cells/hydrogel scaffold), CSD2 (600,000 cells/hydrogel scaffold), and CSD3 (800,000 cells/hydrogel scaffold). Scale bar = 1000 µm. Figure S2: Digital reconstruction of hydrogel’s microgeometry (in light blue) and the seeded spheroids (in orange) under perfusion flow (in dark blue) for fluid flow simulations with CSD1 (400,000 cells/hydrogel scaffold) on day 1 (A1) and day 21 (B1). LBM simulations of (A2, B2) wall shear stress map (Pa) and (A3, B3) Dissolved oxygen concentration map (mol.m^-3^) on day 1 and day 21. Scale bar = 500 µm for all images in this figure. Figure S3: (A1) Red oil staining test comparing the adipogenic differentiation capacity and (A2) Alician blue staining test comparing the chondrogenic differentiation capacity of MSCs-IST and primary MSCs on day 14 of culture. Scale bar = 200 µm. Figure S4: Standardized ALP expression (nmol/min/µg, mean + SEM) throughout the culture period under static conditions showing no activation in the absence of hydroxyapatite in the hydrogel preparation. Cell culture was conducted with CSD1 (400,000 cells/hydrogel scaffold), CSD2 (600,000 cells/hydrogel scaffold), CSD3 (800,000 cells/hydrogel scaffold), and CSD4 (1,000,000 cells/hydrogel scaffold).
