# Supplementary material for: Bone Spheroid Development Under Flow Conditions with Mesenchymal Stem Cells and Human Umbilical Vein Endothelial Cells in a 3D Porous Hydrogel Supplemented with Hydroxyapatite
Source: Gels. 2024 Oct 18;10(10):666. doi: 10.3390/gels10100666 (PMC11506954; doi:10.3390/gels10100666)
Supplement: Supplementary file 1 [file gels-10-00666-s001.zip › gels-3241729-Supplementary Materials.pdf]

Supplementary Material

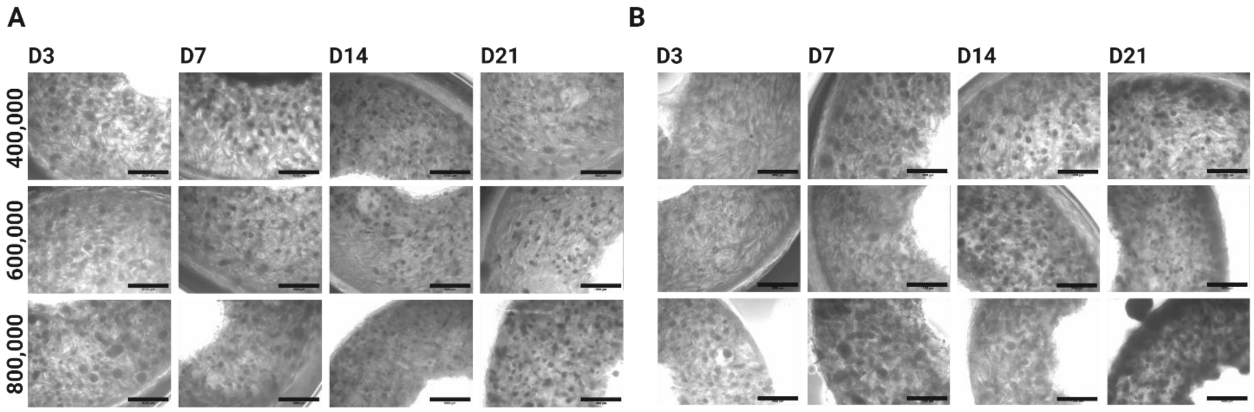

S1: White field acquisitions of seeded hydrogel sections showing the distribution of MSCS/HUVECs spheroids in **(A)** static culture conditions and **(B)** dynamic culture conditions in a perfusion bioreactor. Cell culture was conducted with CSD1 (400 000 cells/hydrogel scaffold), CSD2 (600 000 cells/hydrogel scaffold), and CSD3 (800 000 cells/hydrogel scaffold). Scale bar = 1000 µm.

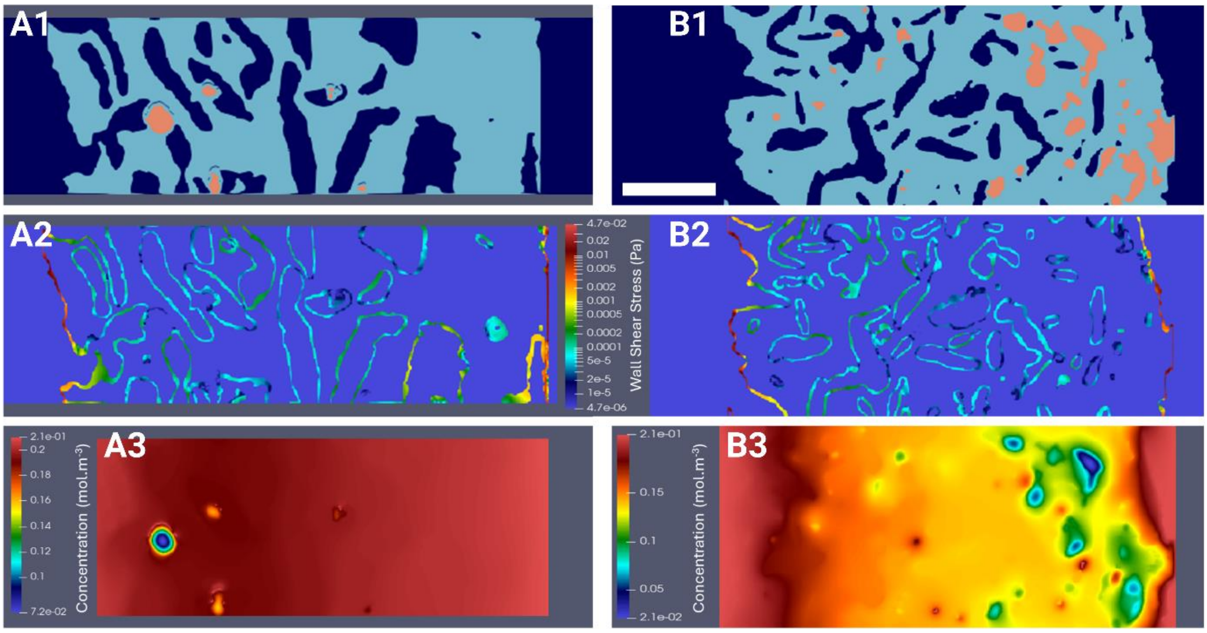

S2: Digital reconstruction of hydrogel's microgeometry (in light blue) and the seeded spheroids (in orange) under perfusion flow (in dark blue) for fluid flow simulations with CSD1 (400 000 cells/hydrogel scaffold) on day 1 **(A1)** and day 21 **(B1)**. LBM simulations of (**A2, B2**) wall shear stress map (Pa) and **(A3, B3)** Dissolved oxygen concentration map (mol.m^{-3}) on day 1 and day 21. Scale bar = 500 µm for all images in this figure.

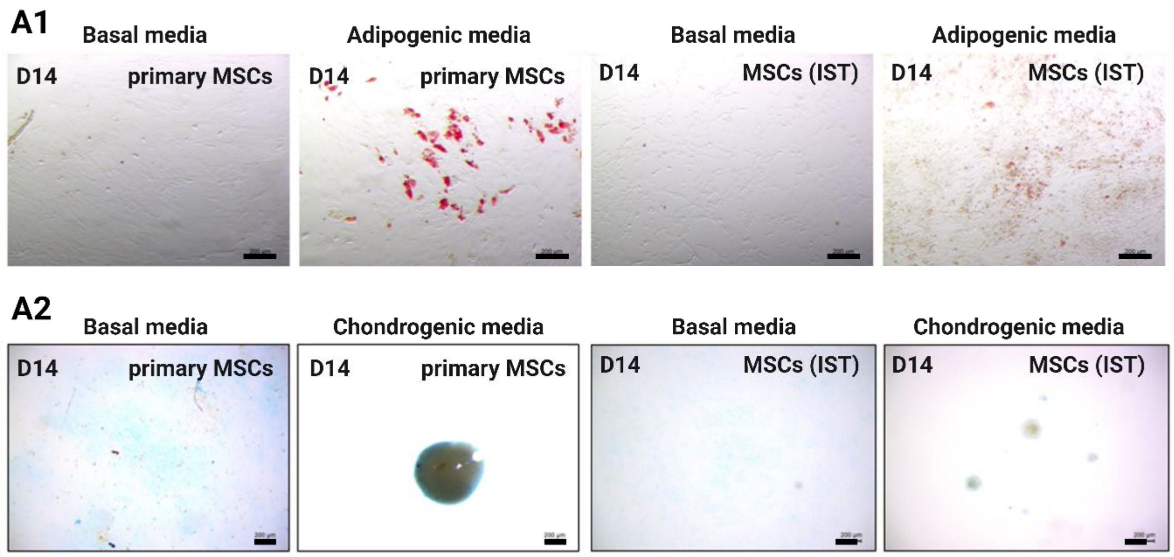

S3: (A1) Red oil staining test comparing the adipogenic differentiation capacity and **(A2)** Alician blue staining test comparing the chondrogenic differentiation capacity of MSCs-IST and primary MSCs on day 14 of culture. Scale bar = 200 µm.

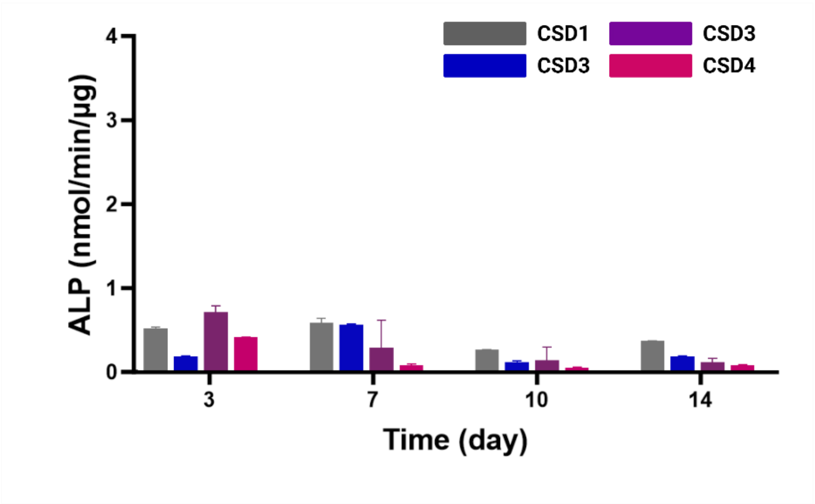

S4: Standardized ALP expression (nmol/min/µg, mean + SEM) throughout the culture period under static conditions showing no activation in the absence of hydroxyapatite in the hydrogel preparation. Cell culture was conducted with CSD1 (400 000 cells/hydrogel scaffold), CSD2 (600 000 cells/hydrogel scaffold), CSD3 (800 000 cells/hydrogel scaffold), and CSD4 (1 000 000 cells/hydrogel scaffold).
